# Primary Non-Hodgkin's Lymphoma of the Scalp and Cranial Vault

**DOI:** 10.1155/2012/616813

**Published:** 2012-03-13

**Authors:** Jovita Martin, Anita Ramesh, Muhamed Kamaludeen, Udhaya, K. Ganesh, Jude J. Martin

**Affiliations:** ^1^Department of Oncology, Sri Ramachandra Medical College, Chennai 600116, India; ^2^Department of Neurosurgery, Sri Ramachandra Medical College, Chennai 600116, India; ^3^Trinity & Co., Saidapet, Chennai 600015, India

## Abstract

Primary Non-Hodgkin's Lymphoma of the cranial scalp and skull vault is a rare disease. We are describing a case of the same in a 50-year-old man. He was presented with a diffuse swelling in the left side scalp since 4 months of duration and progressively enlarging in size. On local Examination of the scalp, there was a diffuse swelling in the left parietal and occipital region of scalp. Imaging showed diffuse infiltration of the skull vault with extracranial soft tissue masses. Further investigations with CT scan chest, abdomen, and pelvis did not reveal any other evidence of systemic lymphoma. Biopsy of one of the scalp masses showed a small to intermediate cell B-cell lymphoma. Other nine previously reported cases of primary skull vault lymphoma were reviewed.

## 1. Introduction

Primary Non-Hodgkin's Lymphoma of the skull with extra- and intracranial extension without systemic or skeletal manifestation in a nonimmunocompromised patient is extremely rare. Till date, only nine such cases have been reported in the literature and in none was the lesion located in the midline [1].

## 2. Case Report

**Figure 1:**
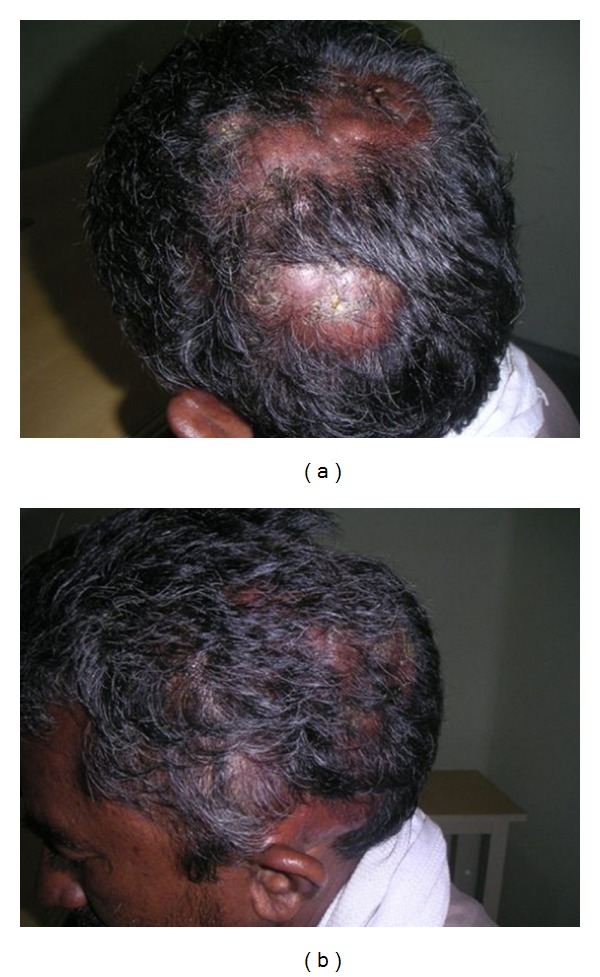
(a) Scalp mass in the left temporoparietal region. (b) Scalp mass in the left temporoparietal region.

 A 50 years male, with 4 months duration of a diffuse swelling in the left side scalp. On local Examination Diffuse swelling in the left parietal and occipital scalp more than 10 × 10 cm, irregular surface, skin over the swelling normal, patchy loss of hair, no tenderness, no transparency or translucency, no pulsation (Figure 1). On general examination there were no other lymph nodes or organomegaly. At that point the clinical diagnosis was soft tissue sarcoma. On blood investigations all the blood parameters were normal, hepatitis B antigen was positive. Bone marrow aspiration study was normal.

**Figure 2:**
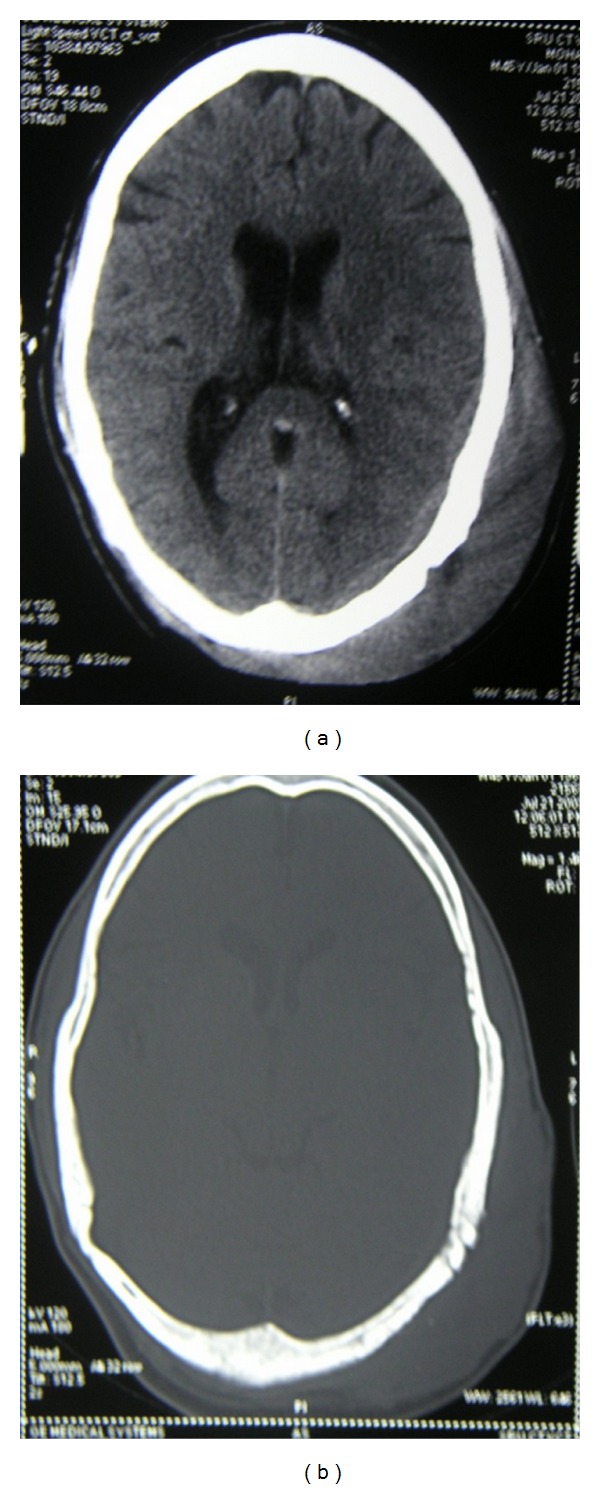
(a) CT scan axial section of the head showing scalp swelling in the left temporoparietal region after partial excision of the mass. (b) CT scan axial section showing scalp swelling after partial excision of the mass.

**Figure 3:**
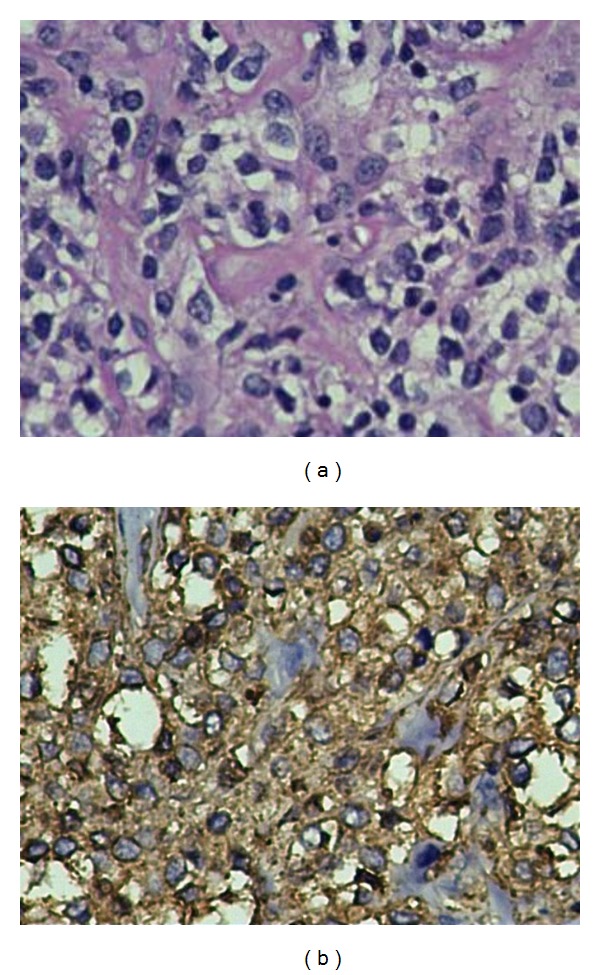
CD 20, CD 45 positivity.

**Figure 4:**
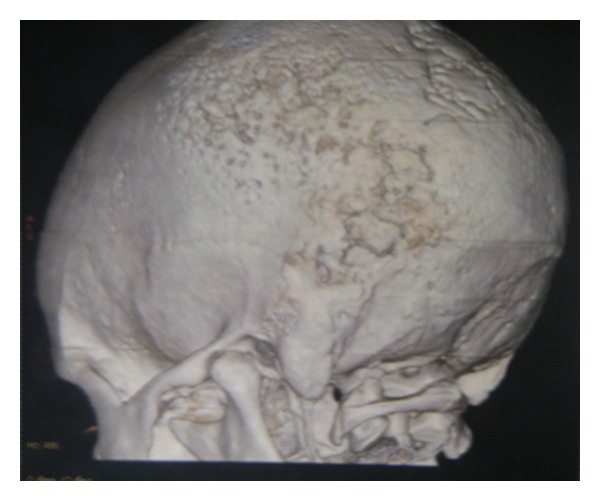
3D reconstructed images of the skull.

 Computed tomographic examination showed Swelling (soft tissue opacity) in the left Parietal region, the surrounding parietal and occipital bones shows bony erosions with cortical thickening and periosteal reaction withmotheaten pattern of destruction.There is associated component of subdural collection (Figures 2 and 4). There was no concurrent nodal or intracranial (extradural/meningeal) involvement or invasion of the orbit. Further staging workup was completed. The CT of the chest and abdomen/pelvis did not reveal any other sites of disease. Histologic examinations revealed diffuse primary cutaneous B-cell lymphoma of the scalp Stage I EA (according to the Revised European-American Lymphoma (REAL) classification). The immunohistochemistry showed CD 20, CD 45 positive (Figure 3).

 The patient was treated with surgery with partial removal of the scalp lesion followed by 6 cycles of chemotherapy with injection cyclophosphamide, doxorubicin, vincristine, and tab. predonisolone. (CHOP regimen) and showed good response with regression of the cutaneous lesions. Local adjuvant External beam Radiotherapy of 36 Gy @ 2 Gy per fraction in 18 fraction for a period of 3 weeks, is planned to be given to the left scalp region was given with 9 MeV electrons.

## 3. Discussion

Most primary cutaneous B-cell lymphomas (PCLs) have been reported involving the head and neck region and therefore PCL of the scalp needs to be differentiated from primary malignant Non-Hodgkin's lymphoma of cranial vault which is more frequently associated with intracranial (extradural) extension [2]. Other important clinicopathologic and radiological differential diagnoses include protruding tumorous angiolymphoid hyperplasia with eosinophilia (ALHE), Kimura's disease of the scalp, pseudolymphoma, cranial vault meningioma, and metastasis, and so forth [3, 4]. The main clinicoradiological differences between PCL of the scalp and primary NHL of the cranial vault include a shorter duration of symptomatology and early onset of focal neurological deficits, large soft-tissue mass, and extensive osteolytic lesions in primary NHL of the cranial vault. As seen in our case, the disease ran an indolent course over a period of one year with no intracranial/orbital invasion and subtle destruction of underlying bones by the diffusely infiltrating soft tissue mass [5]. Notwithstanding, primary malignant lymphoma originating from the skull may extend outside the cranium first and within the cranium subsequently and therefore at presentation more than half of the patients report a scalp mass rather than any neurological sign. Conversely, subcutaneous malignant lymphoma may involve the underlying skull and dura eventually [6, 7]. 

Most PCLs are hyperdense on unenhanced CT and show marked enhancement on postcontrast studies. Magnetic resonance imaging is important for evaluating local and regional spread to see involvement of bone marrow, leptomeninges, or dural venous sinuses [8].

Gallium scanning can rule out or confirm extracutaneous involvement and the presence or absence of activity in cases of gallium avid lymphomas is helpful in indicating the nature of residual soft tissue masses following chemotherapy/radiotherapy. Skin is the second most common site of extranodal lymphoma. Among them, 65% are T-cell lymphomas (mycosis fungoides being the most common form of low-grade malignant peripheral cutaneous T-cell lymphoma), 25% B-cell lymphomas, and about 10% rare variants or nonclassifiable lymphomas. Only 15 to 25% of cutaneous lymphomas show extracutaneous manifestations at time of diagnosis. The prognosis is relatively good, since the average survival time from diagnosis is 12 to 14 years [9]. We are reporting this case because of its rare occurrence. A diagnosis of primary lymphoma of the skull vault should always be considered in the differential diagnosis when such a lesion is encountered in middle aged and elderly patients.
